# Enhanced LTP of population spikes in the dentate gyrus of mice haploinsufficient for neurobeachin

**DOI:** 10.1038/s41598-020-72925-4

**Published:** 2020-09-29

**Authors:** Julia Muellerleile, Aline Blistein, Astrid Rohlmann, Frederieke Scheiwe, Markus Missler, Stephan W. Schwarzacher, Peter Jedlicka

**Affiliations:** 1 Institute of Clinical Neuroanatomy, Neuroscience Center, Goethe University Frankfurt am Main, Frankfurt, Germany; 2 Faculty of Biosciences, Goethe University Frankfurt am Main, Frankfurt, Germany; 3 Institute of Anatomy and Molecular Neurobiology, University of Münster, Münster, Germany; 4 Faculty of Medicine, Justus-Liebig-University Giessen, Giessen, Germany

**Keywords:** Hippocampus, Long-term potentiation, Synaptic transmission

## Abstract

Deletion of the autism candidate molecule neurobeachin (Nbea), a large PH-BEACH-domain containing neuronal protein, has been shown to affect synaptic function by interfering with neurotransmitter receptor targeting and dendritic spine formation. Previous analysis of mice lacking one allele of the Nbea gene identified impaired spatial learning and memory in addition to altered autism-related behaviours. However, no functional data from living heterozygous Nbea mice (Nbea^+/−^) are available to corroborate the behavioural phenotype. Here, we explored the consequences of Nbea haploinsufficiency on excitation/inhibition balance and synaptic plasticity in the intact hippocampal dentate gyrus of Nbea^+/−^ animals in vivo by electrophysiological recordings. Based on field potential recordings, we show that Nbea^+/−^ mice display enhanced LTP of the granule cell population spike, but no differences in basal synaptic transmission, synapse numbers, short-term plasticity, or network inhibition. These data indicate that Nbea haploinsufficiency causes remarkably specific alterations to granule cell excitability in vivo, which may contribute to the behavioural abnormalities in Nbea^+/−^ mice and to related symptoms in patients.

## Introduction

Neurobeachin (Nbea) belongs to the BEACH (beige and Chediak-Higashi) domain-containing protein family whose members are involved in the trafficking of membrane proteins (for review, see^1^). Nbea is highly expressed in endocrine cells and neurons where it is enriched at tubulovesicular endomembranes and in the postsynaptic compartment, and plays a role in vesicle secretion, presumably via regulation of protein kinase A (PKA)^2–4^. Previous research established Nbea as an essential molecule in synapse function because homozygous Nbea knockout mice (Nbea^−/−^) die immediately after birth from breathing paralysis due to a dramatic reduction of evoked synaptic transmission at the neuromuscular junction and in respiratory neurons in the brainstem^5,6^. Despite this dramatic phenotype, the exact role of Nbea is still incompletely understood as its effects are pleiotropic and not all phenotypic observations could be repeated in independent models and assays. While it is widely agreed that complete deletion of Nbea in mice leads to defects of spontaneous and evoked synaptic transmission at excitatory and inhibitory synapses^4–7^, only some of the defects can be attributed to reduced surface levels of postsynaptic AMPA, NMDA and GABA_A_ receptor subunits^4,7–10^. Moreover, while the overall density of asymmetric, presumably excitatory synaptic contacts and synaptic vesicle protein levels are diminished in acute slices from Nbea-deficient embryonic mice^5^, these parameters appear normal in primary neuronal cultures^4,8^. In contrast, the number of excitatory synapses on spines, small protrusions arising from dendritic shafts where most excitatory synapses reside, is reduced in both knockout cultures and brain tissue^8,9^. Additional research identified roles of Nbea in dense-core vesicle function in mouse pancreatic cells and platelets^3,11^ and, possibly, in transcriptional regulation^12^. Collectively, these findings indicate that Nbea is a fascinating but elusive molecule that mandates the use of highly specific techniques to uncover its functions.

Nbea was identified as a candidate gene for autism spectrum disorder (ASD) based on a translocation of the Nbea gene found in a patient with ASD^13^. Deletions of the chromosomal regions on which Nbea resides have also been discovered in other patients with ASDs^14–16^ and developmental/intellectual disability^17^. The Nbea gene is located across a common fragile site, making it more vulnerable to breakage during metaphase^18^. Compared to other genes, the human Nbea gene has a lower tolerance for disruptive mutations, which indicates that it is under purifying selection and thus a likely candidate gene for ASD^19^. To address this important aspect, several groups investigated heterozygous Nbea^+/−^ mice, which exhibit a 30% reduction in forebrain Nbea protein levels^8^, for construct and face validity. In fact, autism-related abnormalities are present in Nbea^+/−^ mice including altered social behaviours, increased self-grooming, delayed spatial learning and memory, increased conditioned fear responses, and impaired fear memory extinction^20,21^. Along with these findings, long-term potentiation (LTP) was enhanced in acute slices of the CA1 region of the hippocampus, an important site for spatial learning^20^. These intriguing results indicate that Nbea might play an important role in the regulation of synaptic plasticity in the hippocampus, but this finding has not been confirmed by in vivo experiments.

Here, we performed electrophysiological recordings in the hippocampus of Nbea^+/−^ mice in vivo to study the potentially important role of Nbea in synaptic transmission and plasticity in the intact brain. Field potentials evoked by stimulation of the perforant path were recorded in the granule cell layer of the dentate gyrus in anaesthetised mice. We found that Nbea^+/−^ mice displayed enhanced LTP of the granule cell population spike, which shows that Nbea has a functional role in vivo that could help explain the behavioural phenotypes observed in haploinsufficient mice.

## Results

### The granule cell population spike is enhanced following LTP induction in Nbea^+/−^ mice

**Figure 1:**
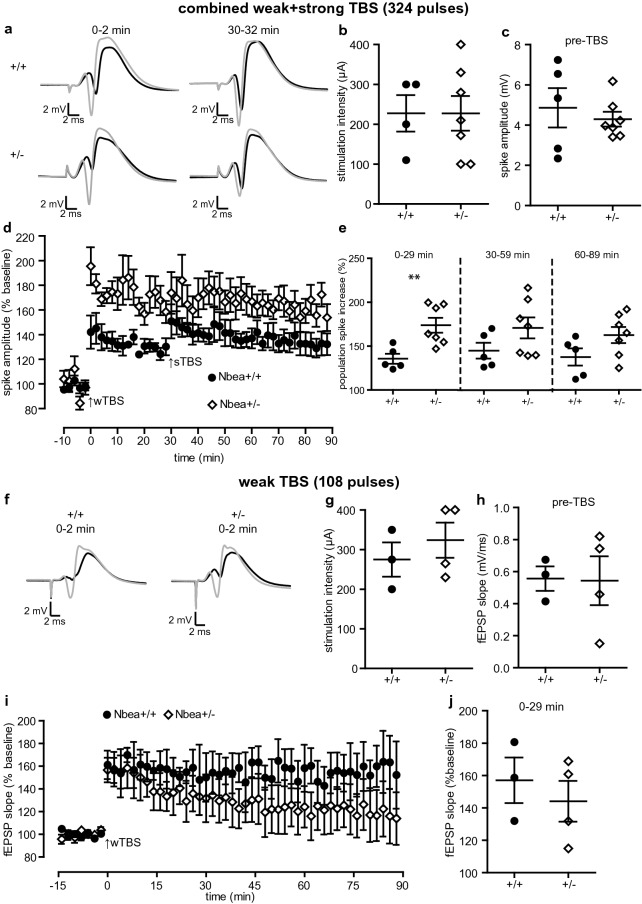
Enhanced long-term potentiation of the population spike at perforant path-to-granule cell synapses in Nbea^+/−^ mice. (**a**–**e**) Long-term potentiation (LTP) of the population spike amplitude induced by weak theta-burst stimulation (wTBS, 3 series of 6 trains of 6 stimuli at 400 Hz, 200 ms between trains and 20 s between series) at 0 min followed by strong TBS (sTBS, 6 series of 6 trains of 6 stimuli at 400 Hz, 200 ms between trains and 20 s between series) at 30 min. (**a**) Representative traces showing the averaged responses during the 2 min preceding (black trace) and the 2 min immediately following (grey trace) wTBS and sTBS for a wild-type and Nbea^+/−^ mouse. (**b**) No difference in the stimulation intensity (P = 0.9991) or the (**c**) pre-TBS population spike amplitude (P = 0.5517) between Nbea^+/−^ mice as compared to wild-type littermate controls. (**d**) Time course of the spike LTP experiments. (**e**) Comparison of the mean increase in the population spike in Nbea^+/−^ and wild-type mice during the 30 min following wTBS (**P = 0.0046), the first 30 min following sTBS (P = 0.1405), and the last 30 min of recording (P = 0.0929). (**f**–**j**) LTP of the fEPSP slope induced by wTBS alone. (**f**) Representative traces showing the averaged responses during the 2 min preceding (black trace) and the 2 min immediately following (grey trace) wTBS for a wild-type and Nbea^+/−^ mouse. (**g**) No difference in the stimulation intensity (P = 0.4767) or the (**h**) pre-TBS fEPSP slope (P = 0.9421) between Nbea^+/−^ mice and wild-type littermate controls. (**i**) Time course of the slope LTP experiments. (**j**) Comparison of the increase in the fEPSP slope following wTBS in wild-type and Nbea^+/−^ mice (P = 0.5302). Unpaired Welch's *t* test used for all comparisons. All values reported as means ± SEM.

Nbea deficiency is known to improve the induction and maintenance of long-term potentiation (LTP) at Schaffer collateral synapses in acute slices of the hippocampus in vitro^20^. Here, we tested whether LTP is also altered in intact animals, a prerequisite for drawing firm conclusions about the pathomechanism in human patients with Nbea haploinsufficiency^13^. LTP at perforant path-to-granule cell (PP-GC) synapses in Nbea^+/−^ and wild-type littermate control mice was induced by theta-burst stimulation (TBS) protocols. Since Nbea^+/−^ mutant mice lack only about 30% of Nbea protein^8^, we decided to use a previously established stimulation protocol^22^ consisting of a weak TBS followed by strong TBS 30 min after the weak TBS to uncover even subtle differences between both groups (Fig. 1a–e). The stimulation intensities were set to elicit a stable population spike and were similar between groups (Fig. 1b, 4 wild-type and 7 Nbea^+/−^ mice, 227.5 ± 45.71 µA vs. 227.4 ± 43.35 µA, unpaired Welch’s *t* test, P = 0.9991). The pre-TBS population spike amplitude was also quite similar between groups (Fig. 1c, 5 wild-type and 7 Nbea^+/−^ mice, 4.861 ± 0.9791 mV vs. 4.292 ± 0.3686 mV, unpaired Welch’s *t* test, P = 0.5517). During the 30 min following the weak TBS, we observed an almost 40% higher potentiation of the population spike relative to the pre-TBS baseline in Nbea^+/−^ compared to control mice (Fig. 1e, 135.6 ± 5.692% for wild-type and 173.8 ± 8.459% for Nbea^+/−^ mice, unpaired Welch’s *t* test, P = 0.0046). The strong TBS further increased the spike amplitude in wild-type mice, thus substantiating the efficacy of LTP induction by TBS in control mice. In contrast, the population spike amplitude was not further enhanced by the second TBS in Nbea^+/−^ mice, revealing that a weak TBS is sufficient to induce maximal potentiation in Nbea^+/−^ mice (Fig. 1d). There was no significant difference between groups in the spike amplitude potentiation following the strong TBS, neither in the first 30 min (171.2 ± 12.02% for Nbea^+/−^, 145.2 ± 9.004% for wild-type, unpaired Welch’s *t* test, P = 0.1405, Fig. 1e) nor the last 30 min (162.8 ± 9.152% for Nbea^+/−^, 137.7 ± 9.792% for wild-type, unpaired Welch’s *t* test, P = 0.0929, Fig. 1e).

In a separate set of experiments, we induced LTP using only the weak TBS protocol in order to determine whether the Nbea^+/−^ mice also displayed an enhancement in synaptic efficacy as measured by the fEPSP slope (Fig. 1f–j). The population spike was minimised to allow for accurate assessment of the initial fEPSP slope. Both the stimulation intensities (Fig. 1g, 3 wild-type and 4 Nbea^+/−^ mice, 275.0 ± 43.30 µA vs. 323.8 ± 44.60 µA, unpaired Welch’s *t* test, P = 0.4767) and the pre-TBS slopes (Fig. 1h, 0.5564 ± 0.07608 mV/ms for wild-type and 0.5433 ± 0.1521 mV/ms for Nbea^+/−^ mice, unpaired Welch’s *t* test, 0.9421) were similar between groups; however, the population spike amplitudes were highly variable and therefore not included in the analysis. Interestingly, there were no significant differences in the potentiation of the fEPSP slope in the first 30 min after wTBS (Fig. 1j, 4 Nbea^+/−^ and 3 wild-type mice, 144.1 ± 12.57% vs. 157.1 ± 14.08%, unpaired Welch’s *t* test, P = 0.5302). Together, these data demonstrate that (1) even a moderate reduction in Nbea protein levels is sufficient to alter long-term potentiation of population spikes in the dentate gyrus of living mice, but that (2) potentiation fEPSP slope is apparently not affected by Nbea.

### Excitatory transmission at PP-GC synapses is unchanged in Nbea^+/−^ mice

Previous studies revealed a strong reduction of evoked glutamatergic postsynaptic responses in the complete absence of Nbea protein in acute slices and primary neuronal cultures of homozygous mice^4,5^, establishing that Nbea is essential for basic synaptic transmission. However, slice recordings of fEPSP slopes from heterozygous neurons were remarkably normal^20^ and spontaneous glutamatergic postsynaptic responses only showed a slight reduction in frequency and rise time but not in amplitude^8^. In order to explore the pre- and postsynaptic properties of haploinsufficient Nbea^+/−^ mice in vivo, we probed the function of excitatory medial PP-GC synapses with different stimulation protocols.

**Figure 2:**
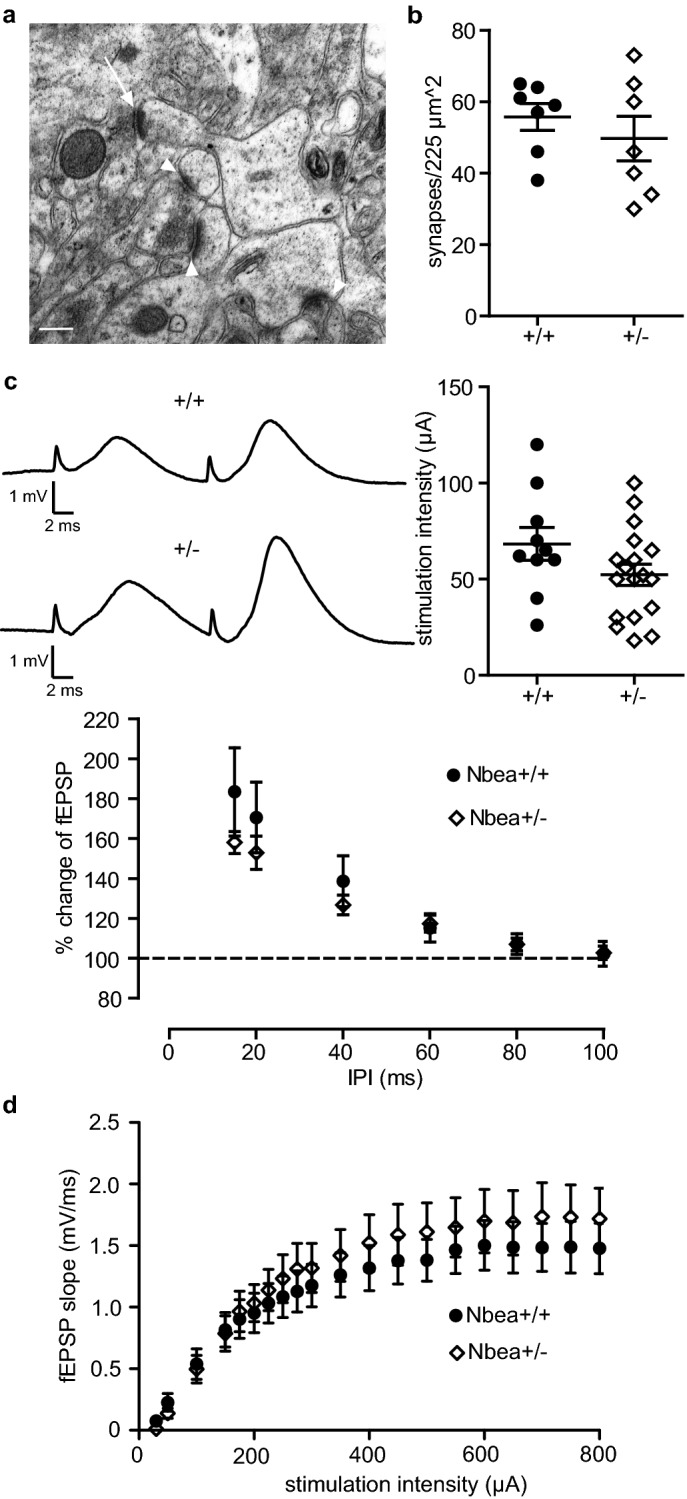
Normal synaptic ultrastructure and basal synaptic transmission at perforant path-to-granule cell synapses in Nbea^+/−^ mice. (**a**) Representative electron micrograph of the middle molecular layer of the dentate gyrus from a Nbea^+/−^ mouse, showing four asymmetric (type 1) synapses terminating on dendritic profiles (arrowheads). One bouton forms a spinous synapse on an unequivocal dendritic spine (arrow). Scale bar, 200 nm. (**b**) Quantitative comparison of the area density of asymmetric synapses in the middle molecular layer of the dentate gyrus from wild-type and Nbea^+/−^ mice (unpaired Welch’s *t* test, P = 0.4295). (**c**) Paired-pulse facilitation of the fEPSP amplitude elicited at stimulus intensities subthreshold for spike generation was not different in wild-type and Nbea^+/−^ mice (two-way ANOVA with Bonferroni's multiple-comparison tests, P = 0.2876 for genotype, P = 0.2434 for interaction). Example traces show the response to PPF stimulation at an interpulse interval (IPI) of 15 ms. Inset: stimulation intensities used to elicit PPF were not significantly different between groups (unpaired Welch’s *t* test, P = 0.1354). (**d**) The response of the fEPSP slope to perforant path stimulation at increasing stimulus intensities shows no difference between wild-type and Nbea^+/−^ mice (two-way ANOVA with Bonferroni's multiple-comparison tests, P = 0.6143 for genotype, P = 0.7891 for interaction). All values reported as means ± SEM.

To exclude the possibility that morphological changes such as a reduction in the number of synaptic contacts interfered with our analysis of functional plasticity, we examined the relevant ultrastructure of these synapses. Samples of the middle molecular layer of the hippocampal dentate gyrus, the very location of medial PP-GC synapses, were imaged in wild-type littermate control and Nbea^+/−^ mice by transmission electron microscopy. No apparent structural alterations were detected concerning the shape of asymmetric (type 1) synapses (representative image in Fig. 2a) or their position on dendritic spine heads and dendritic shafts. Moreover, quantification of the area density of the number of asymmetric synapses in the middle molecular layer revealed no significant differences between control and mutant mice (Fig. 2b, n = 7 sections from 2 mice per genotype, 49.71 ± 6.221 for Nbea^+/−^ and 55.71 ± 3.790 for wild-type mice, unpaired Welch’s *t* test, P = 0.4295).

Next, we measured paired-pulse facilitation (PPF), a form of presynaptic short-term plasticity which depends mostly on the condition of presynaptic terminals^23^. To elicit PPF, we stimulated the perforant path axons at increasing interpulse intervals (IPIs). To avoid activation of the postsynaptic membranes by the firing of granule cells, we used intensities that were subthreshold for population spike evocation. We then measured the augmentation of the fEPSP amplitude (PPF) as the ratio of the two fEPSP amplitudes induced by two successive stimuli at IPIs from 15 to 100 ms (Fig. 2c). As shown previously, PP-GC synapses express PPF at low IPIs which declines with increasing IPI^22,24^. The stimulation intensities (in µA) used to elicit PPF were not significantly different between groups (52.28 ± 5.526 for Nbea^+/−^ and 68.30 ± 8.561 for wild-type, unpaired Welch’s *t* test, P = 0.1354). The variation observed in the degree of PPF was not due to genotype (n = 19 Nbea^+/−^ and 10 wild-type mice, P = 0.2876 determined by two-way ANOVA with Bonferroni's multiple-comparison tests) or the interaction between IPI and genotype (P = 0.2434), suggesting that properties of short-term plasticity are not altered by the reduction in Nbea protein levels.

Furthermore, to exclude the possibility that input–output (I–O) relations were changed in Nbea^+/−^ neurons, I–O curves were measured to probe differences in basal excitatory transmission of PP-GC synapses. We stimulated the perforant path across a range of stimulation intensities (30–800 µA, Fig. 2d) and analysed the slope of the resulting fEPSPs, since the slope is an indicator of synaptic strength^25^. We found that the variation in the I–O relationship was not caused by the genotype (n = 14 Nbea^+/−^ and 10 wild-type mice, two-way ANOVA with Bonferroni's multiple-comparison tests, P = 0.6143), or the interaction between stimulation intensity and genotype (P = 0.7891), confirming results obtained from slice recordings at the Schaffer collateral terminals^20^. Together, these results indicate that a moderate reduction in Nbea protein levels by about 30% in haploinsufficient mice^8^ does not affect properties of basal excitatory transmission at hippocampal synapses in vivo.

### EPSP-spike coupling is slightly reduced in Nbea^+/−^ mice

**Figure 3:**
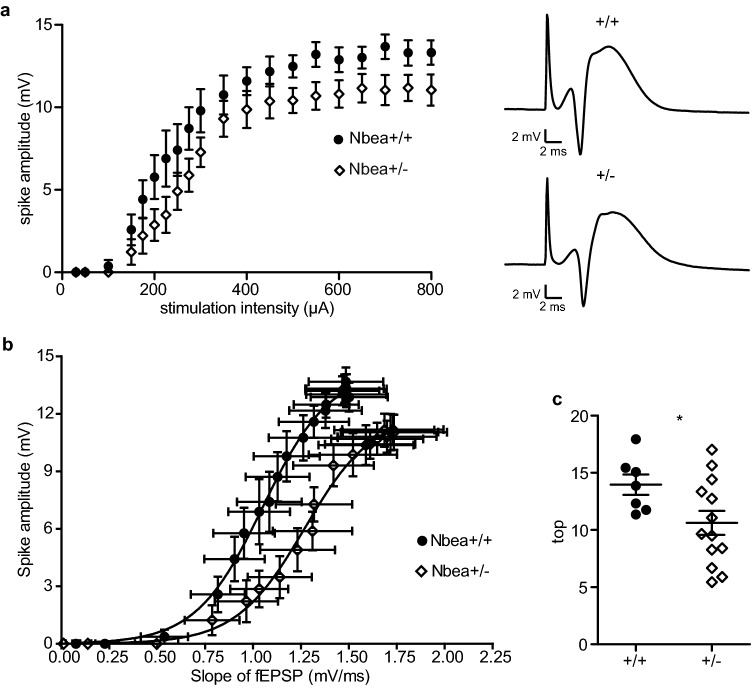
Lower excitability and impairment of EPSP-population spike (E–S) coupling in Nbea^+/−^ mice. (**a**) The input–output curve for the population spike amplitude in response to increasing stimulation intensities was not significantly different when comparing wild-type and Nbea^+/−^ mice (two-way ANOVA with Bonferroni's multiple-comparison tests, P = 0.0676 for genotype, P = 0.4168 for interaction). Representative traces show the responses to 800 µA stimulation. (**b**) EPSP-spike (E–S) coupling fitted with a Boltzmann function. (**c**) At higher stimulus intensities, the coupling between fEPSP slope and population spike values as measured by the top fit parameter is significantly reduced in Nbea^+/−^ mice (unpaired Welch’s *t* test, *P = 0.0261). All values reported as means ± SEM.

One advantage of our recording method is that the synaptic strength and the excitability of the granule cell population can be assessed separately. The population spike is superimposed on the fEPSP and represents the summed firing activity of the granule cells^26^. Therefore, we studied the excitability of granule cells in control versus Nbea^+/−^ mice by measuring the population spike amplitudes across a range of stimulus intensities from 30 to 800 µA. Comparing the results from both groups (Fig. 3a), there were no significant differences in the ability of Nbea haploinsufficient granule cells to produce action potentials compared to their wild-type littermates (n = 14 Nbea^+/−^ and 10 wild-type mice, two-way ANOVA with Bonferroni's multiple-comparison tests, P = 0.0676). The interaction between stimulation intensity and genotype was also not a significant factor in explaining the variation (P = 0.4168). These data suggest that the action-potential-generating capability of GCs is not altered by deletion of one allele of the Nbea gene.

To further examine the relationship between the synaptic input strength and firing ability of granule cells, we performed a fEPSP–population spike (E–S) coupling analysis by plotting fEPSP slopes against population spike amplitudes (Fig. 3b). The coupling between synaptic drive and the action potential firing ability reflects the intrinsic excitability of the neurons^27^ as well as the excitation/inhibition balance of the local circuits^28^. This analysis revealed a rightward shift and a lower top parameter of the Boltzmann-fitted Nbea^+/−^ E–S curve (Fig. 3c, n = 7 wild-type and 13 Nbea^+/−^ mice, 13.96 ± 0.8924 vs. 10.62 ± 1.044, unpaired Welch’s *t* test, P = 0.0261) with respect to the wild-type curve. The rightward shift indicates that heterozygous mice require a stronger synaptic input than their wild-type littermates to produce a similar spike response, i.e. reduced E–S coupling. The reduction in the top parameter reflects the lower maximal spike amplitude in heterozygotes. Thus, our in vivo recordings revealed a reduced tendency of Nbea^+/−^ neurons to translate changes in synaptic input into changes in firing. We therefore tested whether this reduced E–S coupling was due to differences in the balance of excitation and inhibition in the dentate gyrus of Nbea^+/−^ mice by examining network inhibition.

### Network inhibition is unaltered in Nbea^+/−^ mice

**Figure 4:**
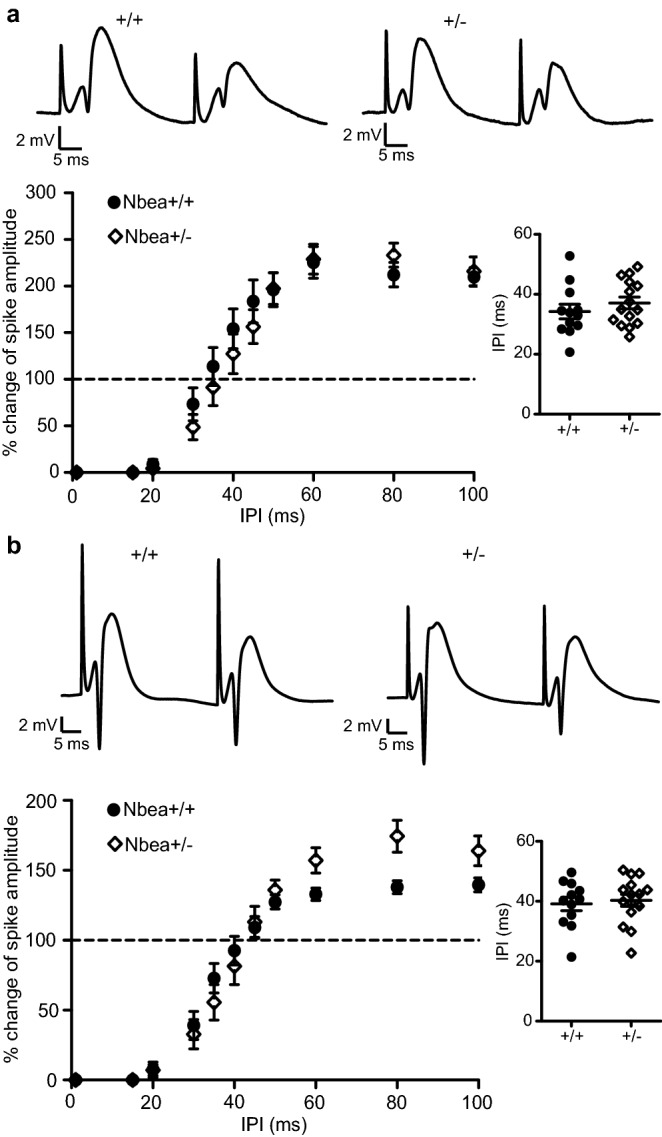
Unaltered network inhibition in Nbea^+/−^ mice. (**a**) Paired-pulse inhibition and disinhibition of the population spike amplitude (PPI/PPDI) at the minimum stimulus intensity for eliciting a spike was not significantly different in wild-type and Nbea^+/−^ mice (two-way ANOVA with Bonferroni's multiple-comparison tests, P = 0.6760 for genotype, P = 0.3239 for interaction). The interpulse interval (IPI) at which PPI switched to PPDI was also not significantly different (inset, unpaired Welch’s *t* test, P = 0.3766). Representative traces show the response to stimulation at 30 ms IPI. (**b**) Paired-pulse inhibition and disinhibition of the population spike amplitude (PPI/PPDI) at the maximal stimulus intensity of 800 µA was not impaired in Nbea^+/−^ compared to wild-type mice (two-way ANOVA with Bonferroni's multiple-comparison tests, P = 0.4264 for genotype, P = 0.0057 for interaction). The IPI at which PPI switched to PPDI was not significantly different (inset, unpaired Welch’s *t* test, P = 0.6948). Representative traces show the response to stimulation at 35 ms IPI. All values reported as means ± SEM.

Recordings in intact mice allow the assessment of distinct properties of neuronal network behaviour that are often difficult to replicate in acute slices or cultures, such as the influence of inhibition on firing activity^29^. Earlier studies have reported that inhibitory synapse function was severely compromised in both acute slices and primary neuronal cultures of null-mutant Nbea neurons^4,5,7,8^. We assessed network inhibition in the dentate gyrus of Nbea^+/−^ mice in vivo by measuring paired-pulse inhibition (PPI) at short IPIs. In addition, we determined disinhibition (PPDI) at longer IPIs. PPI reflects the recruitment of GABAergic interneurons mediating granule cell inhibition by both feedforward and feedback mechanisms^26,30,31^, and PPDI reflects the inhibition of these interneurons^32^. We first applied stimulation intensities triggering population spikes of approximately 1 mV but could not detect significant differences in PPI between Nbea^+/−^ and wild-type mice. PPI/PPDI curves were nearly congruent (Fig. 4a, n = 15 Nbea^+/−^ and 12 wild-type mice, two-way ANOVA with Bonferroni's multiple-comparison tests, P = 0.6760 for genotype, P = 0.3239 for interaction) and the IPI at which PPI switched to PPDI was similar in both groups (37.09 ± 1.937 ms for Nbea^+/−^ and 34.27 ± 2.453 ms for wild-type mice, Welch’s *t* test, P = 0.3766). To determine whether there was a difference between wild-type and Nbea^+/−^ mice at a higher stimulus intensity, we applied 800 μA/0.2 ms stimuli to recruit as much feedback inhibition as possible. The genotype effect was not significant (Fig. 4b, n = 15 Nbea^+/−^ and 12 wild-type mice, two-way ANOVA with Bonferroni's multiple-comparison tests, P = 0.4264), but the interaction between genotype and IPI was significant (P = 0.0057). This indicates that there might have been an effect of the genotype at some IPIs, but there were no major functional consequences to this discrepancy since the PPI/PPDI shift was not significantly different between groups (40.30 ± 1.995 ms for Nbea^+/−^ and 39.11 ± 2.241 ms for wild-type mice, unpaired Welch’s *t* test, P = 0.6948). These results suggest that the haploinsufficiency of Nbea does not primarily manifest itself at inhibitory synapses, although more subtle changes or effects at distinct types of inhibitory neurons cannot be excluded based on our finding of decreased E–S coupling.

## Discussion

Using in vivo field potential recordings from the mouse hippocampus, we provide evidence for an involvement of the neuronal BEACH-domain containing protein Nbea in granule cell excitability in the intact brain. While LTP of the population spike was enhanced in the dentate gyrus of Nbea^+/−^ mice, LTP of the fEPSP slope as well as basal synaptic transmission, short-term presynaptic plasticity, and network inhibition were unaffected. These are important findings because they demonstrate that even a moderate reduction of Nbea protein level^8^ can cause functional abnormalities in distinct neural circuitry.

Our results indicate an enhanced response to LTP stimulation in Nbea^+/−^ mice. Following LTP induction with a weak TBS protocol in vivo, we found a comparable increase in synaptic potentiation and a significantly larger increase in the granule cell spike response to perforant path stimulation in Nbea^+/−^ mice. A previous study reported enhanced LTP in the CA1 region in acute slices from female Nbea^+/−^ mice following a similar TBS protocol^20^, indicating that Nbea plays a functional role in different hippocampal subregions and in different aspects of LTP. What is the behavioural relevance of enhanced LTP? Although LTP is widely believed to be essential for synaptic modifications underlying memory formation, enhanced LTP does not necessarily lead to improved memory formation. In fact, multiple studies have reported an association between impairments in learning and memory and augmented LTP^33–38^. In line with the inverse correlation between spatial memory and LTP, Nbea^+/−^ mice showed impaired learning and memory in behavioural tests^20^. The learning deficits were most pronounced in delayed spatial learning tested in the Morris water maze task, which is highly sensitive to hippocampal lesions^39^. However, Nuytens et al. (2013) only reported one measure of LTP, namely, the EPSP slope. A previous study found a correlation between LTP of the CA1 population spike and the performance of rats in the Morris water maze, whereas LTP of the EPSP was not correlated^40^. This suggests that potentiation of the population spike, and not the EPSP, forms the physiological basis for delayed spatial learning. Furthermore, while both CA1 and the dentate gyrus are important sites for spatial learning^41,42^, the dentate gyrus is also important for the discrimination of similar environments (pattern separation)^43^. In line with this, an increase in LTP has been shown to coincide with less specific hippocampal place fields^44^. During spatial exploration, the capability for enhanced LTP of the population spike may lead to overactivation of the granule cells and hence to a seemingly paradoxical decrease of information content in the hippocampal neuronal network, which may be associated with learning deficits^33^. Therefore, our finding that Nbea^+/−^ mice displayed enhanced population spike LTP in the dentate gyrus can help explain their impairments in spatial memory. From this we predict that future behavioural tests might be successful in revealing deficits in pattern separation tasks in Nbea^+/−^ mice.

Despite the difference in spike LTP, we found that basal synaptic function was unaffected by Nbea deficiency. There were no significant changes in the input–output relationship of fEPSP slopes to the stimulation intensity in Nbea^+/−^ mice as compared to their wild-type littermates. Therefore, the basal glutamate-mediated synaptic transmission is likely not affected by the haploinsufficiency of Nbea. These results are consistent with previous work comparing input–output curves in acute hippocampal slices between wild-type and Nbea^+/−^ mice where no differences were detected at low stimulation intensities^20^. However, previous experiments using Nbea^−/−^ neurons found that in contrast to spontaneous release events, the amplitudes of action-potential induced excitatory postsynaptic currents were significantly affected by the complete lack of Nbea^9^. This can be explained directly by the impaired AMPA receptor subunit trafficking to postsynaptic membranes^9^ which limits the number of glutamate receptors. While the amount of receptors in Nbea^−/−^ neurons might suffice to respond to the neurotransmitter release by single vesicles, they are more than saturated by an action potential induced glutamate release^9^. Unaltered evoked glutamatergic responses in Nbea^+/−^ mice indicate that either AMPAR-subunit trafficking is less impaired in haploinsufficient animals or they have compensatory mechanisms to generate equally strong responses as wild-type mice.

Presynaptic short-term plasticity at perforant path-granule cell synapses was not affected by a reduction in Nbea levels as revealed by unchanged paired-pulse facilitation (PPF). This finding is in line with in vitro experiments using both heterozygous^20^ as well as knockout^4^ neurons. In acute hippocampal slices, PPF at CA1 synapses was not affected^20^. In recordings from synaptically connected pairs of neurons in culture, it was found that the synaptic phenotype of Nbea knockout was only observed if the responding postsynaptic neuron was Nbea-deficient, indicating that presynaptic short-term plasticity mechanisms are unaltered^4^. While these results are seemingly in opposition to the earlier results indicating a strong presynaptic role for Nbea^5,6^, this discrepancy might reflect a difference in Nbea function between hippocampal and cortical neurons where Nbea acts mostly postsynaptically^4,20^ and brainstem neurons^5^ and neuromuscular junctions^6^ where Nbea also has an important presynaptic function.

In contrast to the measures of synaptic transmission, action potential generation (measured by the population spike amplitude) in the granule cells was slightly, but non-significantly, reduced in Nbea^+/−^ mice. The E–S analysis, in which the population spike amplitudes are plotted against the fEPSP slopes for each stimulation intensity, revealed diminished granule cell firing in response to an EPSP of a given size in Nbea^+/−^ mice as compared to wild-type mice, suggesting that Nbea might regulate the intrinsic excitability of granule cells (albeit at very high stimulation intensities that may not reflect physiological levels of synaptic activity). A decrease in E–S coupling could reflect a higher level of feed-forward inhibition in Nbea^+/−^ mice, which is mediated by basket cells^45^ or it could be due to changes in voltage-gated conductances that increase the intrinsic excitability of the granule cells. Further investigations using paired recordings are necessary to answer this question definitively.

To our surprise, we were unable to observe any differences in network inhibition between Nbea^+/−^ and wild-type mice as assessed by PPI/PPDI measurements. This was unexpected since Nbea was shown to have a greater effect on inhibitory than excitatory synaptic transmission in brainstem acute slices, which may result in an increased excitation-inhibition (E/I) ratio^5^. In line with this, other studies implicated Nbea in the trafficking of glycine and GABA_A_ receptors to postsynaptic membranes^4,7,46^ and it was shown that Nbea^−/−^ cells had a 40% reduction in cell surface levels of GABA_A_ receptors^4^. Therefore, we expected PPI to be impaired by a reduction in Nbea. However, most of these previous studies were performed using dissociated neuronal cultures^4,7,46^, where the ratio of GABAergic to glutamatergic cells and the connectivity can differ from the in vivo conditions^47^. Another reason for unchanged network inhibition might be that the loss of one allele in Nbea^+/−^ mice is insufficient to reduce GABA_A_ receptor levels.

Based on our data, we conclude that the increased potentiation of the population spike we observed in Nbea^+/−^ mice is due to changes in the intrinsic excitability of granule cells, rather than synaptic mechanisms. This is suggested by our finding that the increase in the population spike of Nbea^+/−^ mice is greater than the fEPSP slope increase, which was comparable to that of wild-type mice following the single weak TBS protocol. The potentiation of the population spike that cannot be accounted for by the potentiation of the EPSP is known as E–S potentiation^48,49^. Since network inhibition measured by PPI was unchanged in Nbea^+/−^ mice, differences in the intrinsic excitability of granule cells are the likeliest cause for the difference in E–S potentiation. Several mechanisms, such as an increase in the conductance of voltage-gated sodium channels^50^ or a reduction in A-type potassium current^51^ have been proposed to underlie E–S potentiation, though calcium influx appears to be essential^49^. Importantly, the action potential threshold does not necessarily correlate with E–S potentiation^49^, so the difference we observed in (pre-TBS) E–S coupling (which is a reflection of the firing threshold and synchrony) is not an indication of the degree of (post-TBS) E–S potentiation in the same cells. The exact mechanism by which Nbea might affect intrinsic excitability is unknown, but it is conceivable that Nbea might regulate the membrane trafficking of A-type potassium channels via its PH (pleckstrin homology)-BEACH domain in a similar manner to its trafficking of GluA2 AMPAR subunits^9^. Another possibility lies in the function of Nbea as an A-kinase anchoring protein^2^. It was previously shown that A-kinase anchoring proteins interact with Kv4.2 channels, which mediate the backpropagation of action potentials, in order to enhance their surface expression^52^ and that the turnover of Kv4.2 channels is regulated by PKA in distal dendrites of CA1 pyramidal cells^53^. The downregulation of Kv4.2 channels has also been proposed as a potential mechanism for the changes in intrinsic excitability observed after LTP induction in the dentate gyrus in vitro^54^. Thus, Nbea might influence the intrinsic excitability of granule cells by trafficking and/or regulating the turnover of voltage-gated ion channels in dendrites.

ASD is often described as a synaptopathy due to many animal models exhibiting changes in synaptic function^55,56^. However, changes in intrinsic neuronal excitability might have stronger impact on the firing activity of neurons that exhibit high levels of dendritic voltage attenuation such as dentate granule cells^57^. Therefore, our finding that the autism candidate gene Nbea affects the intrinsic plasticity of granule cells highlights the importance of studying non-synaptic mechanisms of plasticity. Further research will be necessary to unravel the precise molecular mechanisms of this proposed function, but the difference in population spike LTP we observed suggests that there are important functional consequences to this difference in excitability.

## Methods

### Animals

Animal experiments were performed with every effort to minimise animal suffering and in accordance with local institutional and governmental regulations regarding the use of laboratory animals both at the University of Münster as approved by the Landesamt für Natur, Umwelt und Verbraucherschutz (LANUV, NRW, Germany) under license numbers 84-02.05.20.11.209 and 84-02.04.2015.A423 and at the University of Frankfurt as approved by the Regierungspräsidium Darmstadt and the animal welfare officer responsible for the institution. All experiments were performed on 2- to 5-month-old male heterozygous Neurobeachin (Nbea^+/−^) mice and wild-type littermate controls. Genotyping and characterisation of this mouse line was carried out as reported previously^5,8^. Electrophysiological measurements and all data analyses were carried out by investigators blind to the genotype.

### Anaesthesia and surgery

Urethane (Sigma-Aldrich, Munich, Germany) solution (1.25 g of urethane in 10 ml 0.9% NaCl solution) was used to anaesthetise the animals with an initial injection (1.2 g/kg body weight) applied intraperitoneally. Supplemental doses (0.2–0.5 g/kg) were injected subcutaneously until the interdigital reflex could no longer be triggered. The body temperature of the animal was constantly controlled through a rectal probe and maintained at 36.5–37.5 °C using a heating pad. For local anaesthesia of the scalp prilocainhydrochloride with adrenalin 1:200,000 (Xylonest 1%, AstraZeneca, Wedel, Germany) was injected subcutaneously at the site of incision. The head of the anaesthetised mouse was placed into a stereotactic frame for accurate insertion of electrodes. The ideal electrode positions were based on a mouse brain atlas^58^ and adjusted according to our previous experience of perforant path stimulation in mice in vivo^22,24^. After drilling the stimulation and recording holes into the skull and removing the dura mater, a bipolar stimulation electrode (NE-200, 0.5 mm tip separation, Rhodes Medical Instruments, Summerland, CA, USA or PBSC1075, 1.0 mm tip separation, FHC, Bowdoin, ME, USA) was lowered into the angular bundle of the perforant path (coordinates: 2.5 mm lateral and 3.7 mm posterior to bregma, 1.8 mm below the brain surface). A glass or tungsten recording electrode was positioned above the suprapyramidal granule cell layer (GCL) of the dentate gyrus (coordinates: 1.0 mm lateral and 1.7 mm posterior to bregma). Borosilicate glass capillaries were pulled using a horizontal puller (DMZ-Universal-Electrode-Puller, Zeitz Instrumente, Martinsried, Germany) and filled with physiological saline solution. The tungsten microlectrodes (TM33B01KT, World Precision Instruments, Sarasota, FL, USA) had an impedance of 0.1 MΩ. The recording electrode was lowered into the tissue in 0.05–0.1 mm increments while monitoring the laminar profile of the response evoked by a 500 µA/0.1 ms stimulus. A positive-going EPSP with a superimposed population spike at a latency of approximately 4 ms indicated that the recording electrode had reached the granule cell layer or hilus of the dentate gyrus and that the stimulation electrode had been correctly positioned in the more medial aspect of the perforant path^59^.

### Stimulation protocols and data analysis

Current pulses (20–800 µA, 0.1–0.2 ms duration) were generated by a stimulus generator (STG1004, Multichannel Systems, Reutlingen, Germany). The recorded field excitatory post-synaptic potentials (fEPSPs) were first amplified (P55 preamplifier, Grass Technologies, West Warwick, RI, USA) and then digitised at 10 kHz for visualisation and offline analysis (Digidata 1440A, Molecular Devices, Union City, CA, USA). The analysis of electrophysiological data was executed using Clampfit 10.2 software (Molecular Devices, Union City, MA, USA) as well as custom Matlab scripts (Mathworks, Natick, MA, USA).

Input–output recordings determine granule cell responses to different stimulation intensities and allow insight into properties of basal excitatory transmission in the dentate gyrus. Three responses were collected and averaged for each stimulus intensity ranging from 30 to 800 µA (0.1 ms stimulus duration). The amplitude of the population spike was defined as the average of the amplitude from the first positive peak to the antipeak and the amplitude from the antipeak to the second positive peak. Only those mice that exhibited a population spike at a stimulation intensity of 300 µA or less were included in the analysis to avoid including mice in which the stimulation electrode was not optimally placed. For the analysis of the slope of the fEPSP, only the early component of the waveform, which is less affected by the population spike, was used. In the analysis relating the fEPSP slope to spike amplitude (E–S plot) each curve was fitted using a Boltzmann function.

In order to investigate the conditions of the presynaptic membrane at perforant path-granule cell synapses, paired-pulse facilitation (PPF) of the fEPSP was measured. To achieve PPF of the fEPSP, two subsequent pulses at an intensity below the population spike threshold (between 20 and 120 µA/0.2 ms), with interpulse intervals (IPIs) varying from 15 to 100 ms were applied. Six paired-pulse responses at each IPI were collected and averaged, and the percentage of facilitation was calculated as relative potentiation of the second fEPSP to the first fEPSP.

Paired-pulse inhibition (PPI) and disinhibition (PPDI) of the population spike were examined to assess the efficacy of inhibition in the hippocampal network. Two pulses at weak stimulation intensities (evoking approximately 1 mV population spikes) were delivered with an IPI ranging from 20 to 100 ms. A total of six paired-pulse responses were recorded at each IPI and averaged. The percentage of change of the population spike amplitude was calculated as the relative change of the spike amplitude following the second pulse in comparison to the spike amplitude following the first one. A Boltzmann equation was used to fit PPI/PPDI curves, hereby obtaining the mean IPI at which the amplitudes of both population spikes are expected to be equal.

As a measure of synaptic plasticity and long-term potentiation (LTP), we compared responses with baseline stimulation prior to theta-burst stimulation (TBS) with responses subsequent to TBS. TBS is an effective LTP induction protocol, as the optimal repetition rate corresponds to the frequency of the hippocampal theta rhythm, an EEG pattern previously related indirectly to memory storage processes^60^. LTP was induced using weak TBS (i.e. three series of six trains of six pulses at 400 Hz, with 0.2 s between trains and 20 s between series) alone or a combination of weak TBS followed by strong TBS (i.e. six series of six trains of six pulses at 400 Hz, with 0.2 s between trains and 20 s between series) 30 min later^22^. Both the pulse width and the stimulus intensity during TBS were doubled in comparison to baseline recordings. The intensity of the baseline stimulus was set to generate a reliable population spike in order to examine the differences in LTP of the population spike. Since the potentiated population spike was often so large as to obscure the initial slope of the fEPSP, we performed another set of experiments in which the baseline stimulation intensity was set to elicit a minimal population spike. The maximum allowable baseline stimulus intensity was 400 µA. The potentiation of the population spike/fEPSP slope was expressed as a percentage change relative to the pre-TBS baseline.

### Histology

Following the electrophysiological recordings, some mice were transcardially perfused in deep anaesthesia with 4% paraformaldehyde (PFA) in 0.1 M PBS. Brains were removed and post-fixed overnight in 4% PFA/0.1 M PBS at 4 °C. After embedding in Agar, 50 µm thick coronal sections were cut with a vibratome (Leica VT 1000 s, Wetzlar, Germany) and stored in cryoprotection solution (30% ethylene glycol, 20% glycerin in PBS) at − 20 °C until further use. Hippocampal sections were stained with 0.1% toluidine blue aqueous solution, differentiated in ethanol, and mounted on glass slides with DPX (all chemicals from Merck, Darmstadt, Germany). Finally, the sections were viewed under a microscope (Olympus BX40, Tokyo, Japan) to verify the correct placement of the stimulation and recording electrodes (Supplementary Figure S1).

### Electron microscopy

Brain tissue from wild-type and Nbea^+/−^ mice was embedded in epon resin (Electron Microscopy Science, EMS, Hatfield, USA). For embedding, anaesthetised adult male mice were transcardially perfused with 25 ml of 2% glutaraldehyde (Roth, Karlsruhe, Germany) and 2% PFA (Merck, Darmstadt, Germany) in 0.1 M PB at 37 °C, and postfixed at 4 °C overnight. Blocks of hippocampal tissue were contrasted in 1% osmium tetroxide for 2 h at room temperature. Following washes with distilled water and dehydrating, tissue was incubated with propylene oxide (EMS) for 45 min, infiltrated with propylene oxide/epon (1:1) for 1 h, in pure epon overnight, and hardened at 60 °C for 24 h. Additional contrasting of thin sections from brains was done on Formvar-coated copper grids with a saturated solution of 12% uranyl acetate and lead citrate.

For direct comparison with electrophysiological analysis, coronal samples containing the middle molecular layer (MML) of the dorsal hippocampal dentate gyrus 1.9 mm posterior to bregma, representing the major termination area of the perforant pathway, were investigated. Ultrastructural analysis was done with a transmission electron microscope (Libra 120, Zeiss) at 80 kV, and images taken with a CCD camera (Tröndle, Moorenweis, Germany). For quantifying the density of asymmetric synapses, tissue areas of 225 µm^2^ were reconstructed from 9 individual images and 7 of these reconstructions (total area of 1575 µm^2^) analysed per genotype (n = 7 reconstructions from 2 animals per genotype). Asymmetric (type 1) synapses were defined as contacts with a visible synaptic cleft, a distinct postsynaptic density and at least three synaptic vesicles.

### Statistical analysis

All statistical analyses were performed using the software Prism 7 for Windows or Mac (GraphPad Software, La Jolla, CA, USA). Electrophysiological and ultrastructural data were tested for statistical significance using an unpaired Student's *t* test (with Welch’s correction if the variance or group sizes differed) or a two-way analysis of variance (ANOVA) with Bonferroni's multiple-comparison post-tests. The Shapiro–Wilk test was used to determine whether the samples were drawn from a normal distribution. A two-tailed P-value lower than 0.05 was considered to be significant. Group values are reported as means ± standard error of the mean (SEM).

## Supplementary information

Supplementary Figure.

## Data Availability

All data will be made available upon request.
