# Supplementary material for: Enhanced LTP of population spikes in the dentate gyrus of mice haploinsufficient for neurobeachin
Source: Sci Rep. 2020 Sep 29;10:16058. doi: 10.1038/s41598-020-72925-4 (PMC7524738; doi:10.1038/s41598-020-72925-4)
Supplement: Supplementary file 1 — Supplementary Figure. [file 41598_2020_72925_MOESM1_ESM.pdf]

Supplementary Information

Journal: *Scientific Reports*

Article: Enhanced LTP of population spikes in the dentate gyrus of mice haploinsufficient for neurobeachin

Authors: Julia Muellerleile[1,2,*], Aline Blistein[1,*], Astrid Rohlmann[3], Frederieke Scheiwe[3], Markus Missler[3], Stephan W. Schwarzacher[1,#], Peter Jedlicka[1,4,#]

[1]*Institute of Clinical Neuroanatomy, Neuroscience Center, Goethe University Frankfurt, Frankfurt/Main, Germany*

[2]*Faculty of Biosciences, Goethe University Frankfurt, Frankfurt/Main, Germany*

[3]*Institute of Anatomy and Molecular Neurobiology, University of Münster, Münster, Germany*

[4]*Faculty of Medicine, Justus-Liebig-University Giessen, Giessen, Germany*

[#]*Joint senior authors*

**Corresponding authors (contributed equally to this work):*

Julia Muellerleile
Institute of Clinical Neuroanatomy
Neuroscience Center
Goethe University Frankfurt
Theodor-Stern Kai 7
60590 Frankfurt/Main
Germany
Email: muellerleile@med.uni-frankfurt.de

Aline Blistein
Institute of Clinical Neuroanatomy
Neuroscience Center
Goethe University Frankfurt
Theodor-Stern Kai 7
60590 Frankfurt/Main
Germany
E-mail: aline_blistein@web.de

FIGURE S1: Histological verification of stimulation and recording sites. Nissl-stained sections showing the recording electrode position in the hilus of the dentate gyrus (DG) (top left) and the stimulation electrode placement in the angular bundle of the perforant path (bottom left) in one mouse. Arrows indicate the electrode tracks. Anatomical regions were labeled using a mouse brain atlas (reprinted with permission from *The mouse brain in stereotaxic coordinates*, K. B. J. Franklin & G. Paxinos, Figures 45 and 61, Academic Press, 2001). Abbreviations: CA1=cornu ammonis 1, DG=dentate gyrus, gcl=granule cell layer, hf=hippocampal fissure, dhc=dorsal hippocampal commissure, ml=molecular layer, py=pyramidal cell layer, slm=stratum lacunosum moleculare, so=stratum oriens, sr=stratum radiatum.

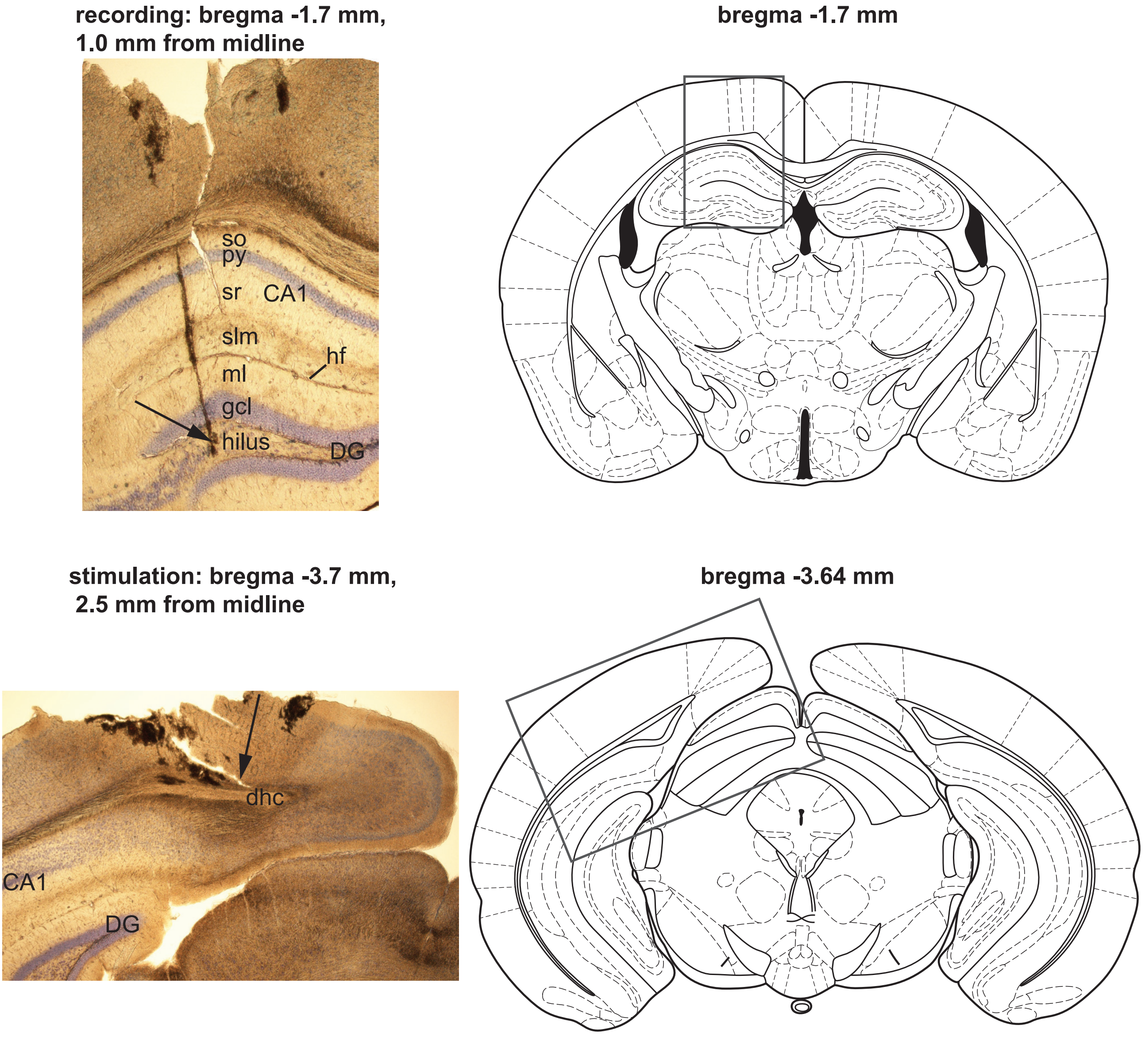
recording: bregma -1.7 mm,
1.0 mm from midline
so
py
sr
CA1
slm
hf
ml
gcl
hilus
DG
bregma -1.7 mm
stimulation: bregma -3.7 mm,
2.5 mm from midline
dhc
CA1
DG
bregma -3.64 mm
